# Treating type-1 diabetes with an epigenetic drug

**DOI:** 10.7554/eLife.05720

**Published:** 2014-12-30

**Authors:** Yohko Kitagawa, Naganari Ohkura

**Affiliations:** Department of Experimental Immunology, World Premier International Immunology Frontier Research Center, Osaka University, Suita, Japan; Department of Experimental Immunology, World Premier International Immunology Frontier Research Center, the Department of Frontier Research in Tumor Immunology, Graduate School of Medicine, and the Center of Medical Innovation & Translational Research, Osaka University, Suita, Japannohkura@ifrec.osaka-u.ac.jp

**Keywords:** autoimmune diabetes, bromodomain inhibitor, NF-KB, mouse

## Abstract

A single drug treats type-1 diabetes in mice by dampening inflammation and enhancing insulin production.

**Related research article** Fu W, Farache J, Clardy SM, Hattori K, Mander P, Lee K, Rioja I, Weissleder R, Prinjha RK, Benoist C, Mathis D. 2014. Epigenetic modulation of type-1 diabetes via a dual effect on pancreatic macrophages and β cells. *eLife* **3**:e04631. doi: 10.7554/eLife.04631

**Image** The drug I-BET151 acts on β cells (red) and macrophages (green) in the islets of the pancreas to treat type-1 diabetes in mice (Image credit: James F Mohan)

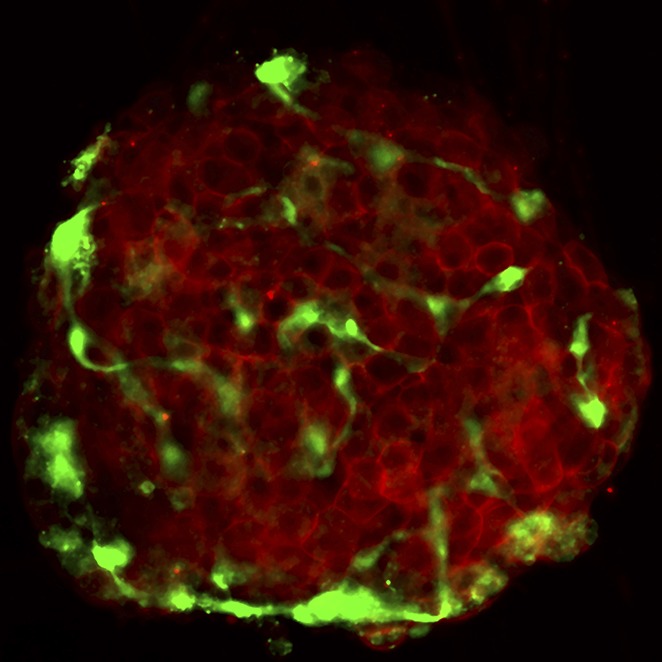

Type-1 diabetes is an autoimmune disease in which the body's own immune cells attack islet β cells, the cells in the pancreas that produce and release the hormone insulin. Chronic inflammation in the islets (called insulitis) eventually leads to the complete destruction of the β cells, resulting in an insulin deficiency that causes type-1 diabetes. Currently, patients with type-1 diabetes must rely on regular injections of insulin to prevent their blood sugar level from rising too high (a condition called hyperglycaemia). As such, there is great demand for the development of effective treatments that correct the underlying problems that cause type-1 diabetes.

Now in *eLife*, Diane Mathis, Christophe Benoist and co-workers at the Harvard Medical School and GlaxoSmithKline—including Wenxian Fu as first author—report that a drug called I-BET151 can effectively prevent type-1 diabetes in a mouse model for this disease. The mice spontaneously develop type-1 diabetes when they are still relatively young. Fu et al. show that in addition to preventing insulitis, treating these mice with I-BET151 also reverses pre-existing insulitis, thereby inhibiting the progression of the disease into type-1 diabetes (Fu et al., 2014).

A number of cell types are involved in the development of type-1 diabetes. These cells include: pathogenic T cells that recognise β cells as a target for destruction; antigen-presenting cells that activate the pathogenic T cells; macrophages that release pro-inflammatory molecules such as cytokines; and β cells that respond to these immunological attacks (Eizirik et al., 2009). There are also several potential therapeutics that target some of these disease-causing pathways, for example by neutralizing the cytokines, or by blocking the interaction of T cells and antigen-presenting cells. Other potential treatments that target the causes of type-1 diabetes include promoting the survival and regeneration of β cells, and converting pathogenic T cells into another type of T cell.

In complex diseases like type-1 diabetes, targeting multiple pathways by combining different drugs is thought to offer a more effective way to prevent the disease from progressing while keeping side-effects to a minimum (Matthews et al., 2010). Indeed, the remarkable efficacy of I-BET151 for the treatment of insulitis is attributed to its dual effects on pancreatic macrophages and β cells. While this drug alters the way that macrophages in the pancreas behave in a way that dampens the local inflammation, it also promotes the proliferation of β cells and restores the tissue's function (Figure 1).

**Figure 1.**
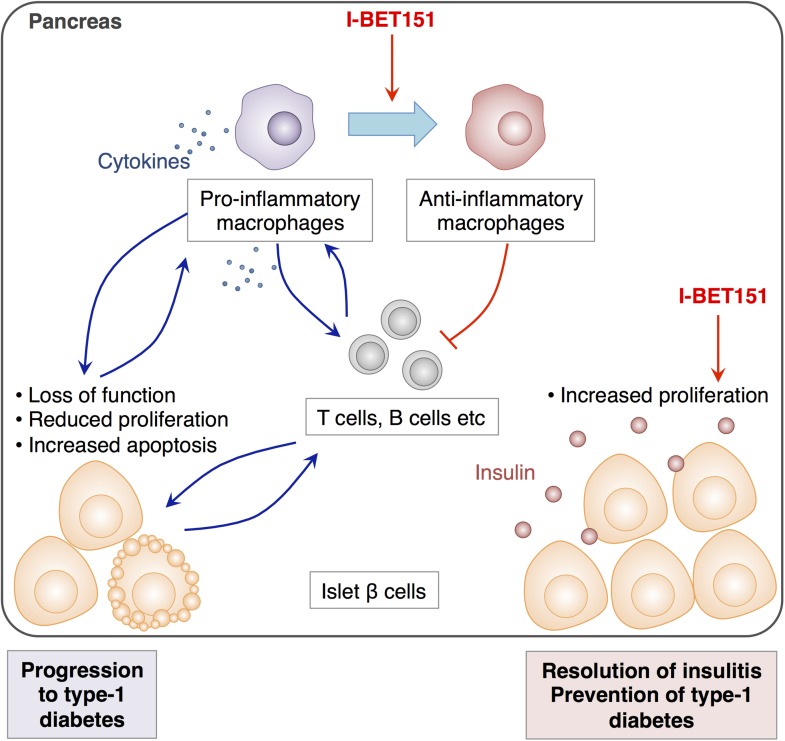
The effects of I-BET151 on the resolution of insulitis and the prevention of type-1 diabetes. Progression to type-1 diabetes is characterised by chronic inflammation and a loss of function of islet β cells (shown in pale orange). This reduces the proliferation of these cells and often leads to a form of programmed cell death called apoptosis (bottom left). Fu et al. reveal that I-BET151 works to resolve inflammation of the islet β cells (insulitis) mainly by two mechanisms. Firstly, it encourages macrophages in the pancreas to convert from being pro-inflammatory (which release cytokines that promote inflammation, top left) to being anti-inflammatory type (top right). This inhibits the further recruitment of T cells and dampens inflammation. Secondly, I-BET151 enhances the proliferation of islet β cells and enhances insulin production (bottom right).

I-BET151 is a synthetic compound, originally generated as a reagent to target the epigenetic codes of the genome (Nicodeme et al., 2010). These codes are chemical modifications on the histone proteins that DNA wraps around, and also on the DNA itself, and they indicate whether expression of a particular gene should be switched on. One type of epigenetic modifications is the acetylation of histones, which is associated with active gene expression. By mimicking the structure of acetylated histones, I-BET151 can interfere with the recognition of acetylated histones by a protein called Brd4, and inhibit the subsequent gene activation. Additionally, other similar Brd4 inhibitors have also been reported to interrupt the binding of Brd4 to an acetylated protein that is a subunit of a transcription factor called NFkB. These inhibitors also repress the expression of inflammation-associated genes, which are normally activated by NFkB (Huang et al., 2009; Zhang et al., 2012; Zou et al., 2014).

To understand the effects of I-BET151 in the course of disease treatment, Fu et al. compared the change in gene expression levels in immune cells caused by administering I-BET151, against the gene expression profiles from over 200 immune cell populations. This comparison elegantly demonstrated that macrophages are the main target of I-BET151. Upon its administration, macrophage reduced the expression of genes that are associated with accelerating inflammation, which was primarily attributed to it inhibiting NFkB-dependent gene activation. Furthermore, I-BET151 increased the expression of molecules with anti-inflammatory properties. In contrast, the effects of I-BET151 on the gene expression of T cells and other immune cells were unexpectedly small. However, chronic inflammation often involves multiple molecules or cells that together act to escalate the inflammatory response. Macrophages and T cells act in this way in the pancreatic tissue of diabetic mice (Yoon et al., 1998; Cantor and Haskins, 2007). Moreover, targeting macrophages using I-BET151 was associated with the reduction in the number of T cells that infiltrate in the tissue, likely contributing to the reversal of insulitis (Fu et al., 2014).

Another critical aspect of the development of type-1 diabetes, along with the inflammation caused by immune cells, is the response of the β cells. Attack by the immune system suppresses the function of the β cells and can cause them to undergo a form of programmed cell death called apoptosis. This, in turn, can further stimulate an immune response (Eizirik et al., 2009). Fu et al. show that I-BET151 enhances the proliferation of β cells in addition to preventing further immunological attacks. This implies that as long as there are functional β cells left, this drug can reverse the disease progression.

With the ability to simultaneously target macrophages and β cells in a cell type-specific manner, I-BET151 appears to be a promising therapeutic drug for the treatment of type-1 diabetes. Whether its effect falls into a category of general immunosuppression, with the possibility of compromising necessary immune responses when applied to humans, will need to be addressed before this drug can be used clinically. It will also be important to determine if there is an appropriate dosage of I-BET151 that treats type-1 diabetes without causing unnecessary epigenetic and transcriptional changes.
